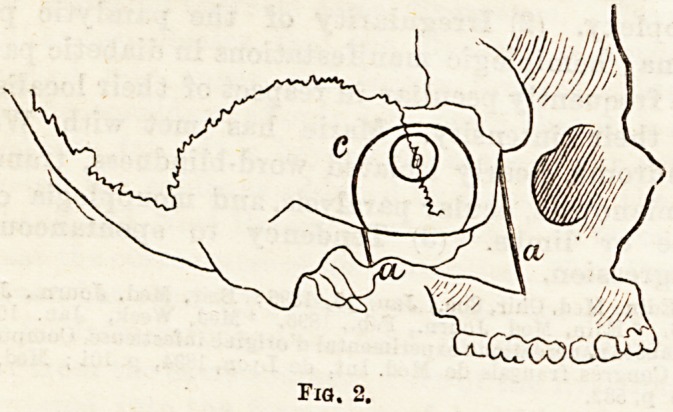# Progress in Surgery

**Published:** 1896-05-02

**Authors:** 


### PROGRESS IN SURGERY.

### SURGERY OF THE NERVES.

**Operations for Trifacial Neuralgia.** — Nothing but the excessive bodily pain and mental anxiety endured by the unfortunate sufferers from persistent trifacial neuralgia would appear to justify the severe operations which are undertaken for their relief. The earlier operations (Rose and Navaro) for dividing the branches of the fifth nerve inside the skull, aimed at reaching them through the base of the skull in the vicinity of the foramen ovale, access being obtained by a preliminary excision of the upper jaw or temporary resection of the malar bone. Later operators (Horsley, Hartley, and Krause) approached the nerve roots from the temporal fossa, removing part of the cranial wall above the zygoma.

I. Doyen,[1] of Rheims, not satisfied with the freedom of access gained by this operation, has practised a more extensive one by the same route, which consists in (1)

a sickle-shaped incision (Fig. 1, *a*) through the soft parts in the temporal region, avoiding as far as possible the branches of the facial vessels and nerve; (2) resection of the zygomatic arch (Fig. 2, *aa*), division of the coronoid process, and denudation of the temporal fossa; (3) the inferior dental and lingual nerves are identified, divided, and their ends secured with toothed

Fig. 1.

forceps; (4) the internal maxillary artery is ligatured close to its point of origin; (5) the skull is opened by a trephine applied over the spheno-temporal suture (Fig. 2, *b*) and the opening is enlarged with gouge forceps until all the lower part of the temporal fossa is removed (Fig. 2, *c*), and this process is carried on until sufficient of the base of the skull is removed to expose the foramen ovale; (6) through the large opening thus made the divisions of the nerve can be freely and certainly divided, and the Gasserian ganglion itself removed. Doyen's results are not brilliant. The

Fig. 2.

first patient recovered; the next two died, one four days after operation, the other, of cerebral apoplexy, ten days after operation.

II. The results of Krause's operation appear to be better than those obtained by Doyen. Krause himself had five cases, with one death. Czerney, of Heidelberg,[2] has recently published three cases where Krause's method was followed, the bone being divided with a circular saw as far as the diploe and completed with a fine chisel. His ultimate results were excellent, in spite of serious complications arising during the after-treatment in two of them. (1) A woman, thirty-seven; neuralgia of seven years' duration; second and third divisions of trifacial divided. Severe and recurring hæmorrhage from the middle meningeal prevented the operation being completed till the fifth day after the first attempt. Later, otitis media (attributed to vaso-motor disturbances) ensued, but disappeared in a fortnight. Six weeks after operation necrosis of a portion of the squamous temporal was associated with feverishness, headache, and vomiting, and followed by a hernia cerebri, which was resected. The patient nevertheless recovered, and a year later was quite free from pain. (2) Man, sixty-four; neuralgia of second division for fifteen years. Hæmorrhage from the middle meningeal during operation, and a secondary hæmorrhage eleven days after interfered with the convalescence. Two years later he was free from pain, and had in the interval a carcinomatous tumour removed from his bladder. (3) Man, twenty-four, second and third divisions involved for four years. Slight hæmorrhage from a branch of the middle meningeal during operation; complete disappearance of pain, but paralysis and atrophy of the muscles of mastication followed.

Gerster[3] reports a cure in the case of a woman, thirty-four, who had suffered from neuralgia of the second division for seven years. He found Krause's operation easy, and the convalescence was uneventful, but for a slight retention of serous fluid owing to the capillary gauze drain failing to act perfectly. The same operator had a fatal case, due to extensive encephalitis. Abbe[3] had two successful cases, in one of which the Gasserian ganglion was removed by a curette. Willy Meyer's[3] case died of tempero-sphenoidal abscess opposite the operation wound four months after operation. In regard to the results of these operations, it may be observed that B. von Beck[4] has collected forty-one cases published since 1890, with six deaths.

III. Some investigations have recently been carried out by Professor Ferrier and Dr. W. Aldren Turner,[5] on the so-called "trophic" influence of the fifth nerve on the cornea, and the results of its section. The conclusions arrived at are that "there is no evidence of trophic influence exerted by the Gasserian ganglion upon the cornea; and that, provided septic organisms are excluded, the ophthalmic branch may be safely divided, or the Gasserian ganglion removed without fear of the disorganisation of the eye." Two cases cited show that even after division of the main trunk behind the ganglion, or of the ophthalmic branch alone, healthy nutrition and even repair may go on, notwithstanding the anæsthetic state of the cornea. Clinical experience bears out these experimental observations, because interference with nutrition of the eye leading to its destruction is exceedingly rare after operations for trifacial neuralgia, and when it does occur can usually be traced to some direct irritative cause.

IV. In an interesting paper,[6] Dr. T. F. Walsh, of Camden, N.J., reports a case of neuralgia of the tongue, treated by stretching the lingual branch of the fifth nerve. The patient, a young woman, had suffered for five years from a sharp shooting pain along the right side of her tongue, the attacks gradually increasing in frequency and severity until extreme pain became almost constant. Local and general anti-neuralgic remedies gave but slight and temporary relief. The operation consisted in making an incision along the lower third of the posterior part of the side of the tongue, exposing the lingual nerve, and stretching it with a force equal to four pounds for about five minutes. This only gave relief for three days, when the pain returned. The nerve was again exposed, and stretched with greater force, with the result that the pain disappeared, and when she was seen seven years later had never returned. During the after treatment of this case observations were made regarding the anatomico-physiological aspects of the lingual nerve, and the following deductions were drawn: (1) The lingual is largely concerned in furnishing taste and sensation to the tongue; (2) it partly supplies the lingualis muscle with motor power; (3) at the tip of the tongue there is a commingling of the terminal filaments of the two lingual nerves, or an anastomotic distribution from the hypoglossal; (4) the glosso-pharyngeal furnishes sensation to the posterior fourth of the tongue, and alone supplies the circumvallate papillæ with taste; (5) the filiform papillæ are specially tactile, the intervening mucous membrane has only ordinary sensation; (6) to the fungiform and circumvallate papillæ belong the sense of taste; (6) the filiform and fungiform papillæ are supplied by the lingual nerve, the circumvallate by the glosso-pharyngeal; (7) the anterior three-fourths of the mucous membrane is supplied by the lingual and the posterior fourth by the glosso-pharyngeal nerve.

**Suture of Musculo-spiral Nerve.**—V. Sinkler[7] reports a case in which he successfully sutured the musculo-spiral nerve three months after its complete division by a stab. There was complete drop wrist and reaction of degeneration, with but little loss of tactile sensibility. It was five months after the nerve was sutured before improvement set in, during which time galvanism was constantly applied.

[1] Archiv. Provinciales de Chirurgie, vol. iv., No. 7. [2] Med. Week, Oct. 18, 1895. [3] Ann. Surg., Jan., 1896. [4] Beitrage z. Klin. Chir., xiii., 3. [5] Brit. Med. Journ., Nov., 1895. [6] Ann. Surg., Jan., 1896. [7] Internat. Med. Mag., Dec., 1895.

### DISEASE OF THE UPPER AIR PASSAGES.

**Accessory Sinuses of the Nose.**—Notwithstanding the large number of recent contributions on antral empyema, there is a great deal yet to learn about the symptoms and causes of this disease; but when we turn to the consideration of frontal, ethmoidal, and sphenoidal sinusitis, we find ourselves continually losing our bearing, and substituting for definite means of diagnosis and definite lines of treatment more or less groping in the dark. We will note recent articles on sinusitis in the following order: *Frontal* and *Ethmoidal, Sphenoidal*, and *Maxillary sinusitis.*

**Frontal and Ethmoidal Sinusitis.**—Bryan,[1] in a very valuable paper, records that he has met with a number of cases of sinusitis following influenza, and his experience in this respect is corroborated by many other observers. It is, however, impossible to determine in what proportion the several accessory sinuses are involved; probably all are equally involved, but the ultimate issue in recovery or suppuration depends on their relative position, and on the different anatomical relations which render drainage more or less difficult. Herzfeld,[2] at autopsies on ten patients who had succumbed to influenza, found acute inflammation of the mucous membrane of the maxillary, frontal, and sphenoidal sinuses. But he states that traumatism, scarlet fever, measles, typhoid fever are frequently associated with suppuration of these cavities. Again, Bryan states that, aside from the true influenza, the inflammation of a simple rhinitis frequently extends into one or more of these cavities, and unless recognised and treated early is most likely to result in an abscess, it being important that these cases be treated early, for upon their early recognition depends their final issue, which in many cases has resulted in the death of the patient. But, aside from acute inflammations, there are other and still more frequent causes, viz., chronic catarrhal rhinitis, hypertrophic rhinitis, polypi, foreign bodies blocking up the middle meatus, so that the secretions are retained within the sinuses, and finally give way to the formation of pus.

Among the earlier symptoms of abscess of the ethmoidal cells, Bryan[3] mentions pain of neuralgic character, referred to the bridge of the nose, increasing in intensity with the progress of the disease, and extending outwardly along the infra-orbital and occasionally along the supra-orbital ridge. With a distension of the cells there is a sense of pain and pressure felt in the orbit, exophthalmia, a narrowing of the fields of vision, and, if there is softening of the bone at the inner angle of the orbit, crepitation may be present. In chronic ethmoidal sinus suppuration, Ziem[4] recommends, firstly, removing polypi and other local causes of obstruction, and secondly, spraying with a spray the jets of which direct the fluid upwards; he prefers to use normal salt solution. Further, the frequent inunction at night of the external nose, its roots and sides, as far as the inner angle of the eye, favourably influences the condition. This method, borrowed from the people, acts principally by preventing the escape of moisture by the skin, causes increased flow towards the mucosa, and thinning of the secretion. When suppuration is in the anterior cells, the treatment may be by the method of Jansen and Kuhnt, viz., by reaching the pus through the opened frontal sinus. When the middle and posterior cells are the seat of suppuration, they may be reached through the orbit, *i.e.*, by a route through which these suppurating sinusites sometimes burst. As regards the frontal sinus, abscess, Bryan finds, may exist without giving rise to any symptoms except a slight discharge of pus from the nose, as in the case reported by Luc,[5] but in most cases the symptoms are pronounced, and vary in intensity according to whether the fronto-nasal duct is open or closed. Pain in the frontal region, at first dull, and then becoming lancinating in character as the secretions distend the cavities, is the most common symptom. There is pain on pressure over and under the supra-orbital ridge; there may be also some redness and swelling of the skin over the affected sinus, which sometimes extend down and involve the corresponding upper eyelid. If the fronto-nasal duct be closed, there is dilatation of the sinus, with a tendency to bulge at its thinnest part —*i.e.*, at the inner angle of the orbit on a level with the root of the nose—and it occasions a displacement of the eye forward, downward, and outward. If there is no relief, the pus finds its way through this swelling into the orbit in the form of an orbital abscess, or ruptures posteriorly into the cranial cavity. Besides the possibility of an orbital abscess forming, among the other eye complications may be mentioned, changes in the corresponding papilla generally a hyperæmia associated with dilated and tortuous veins and a narrowing of the field of vision.

While inflammation and abscess generally extends to the ethmoidal cells, or, by escaping from the opening of the fronto-nasal duct, into the maxillary sinus, it sometimes happens that the maxillary sinus directly communicates with the frontal sinus, either by an anomalous anatomical communication or by a communicating sinus formed pathologically by the extension of the abscess through caries of the anterior ethmoidal cells. This anomaly has also been noted by others; the late Professor Leidy had met with it two or three times, while Curnow (cited by Macdonald) has also met with the same condition.

[1] Rep. Trans. Amer. Laryng. Assoc. xvii. meeting. [2] Rep. of Berlin Med. Soc.; Med. Week. Nov. 22, 1895. [3] Loc. cit. [4] Journ. of Laryng., Dec., 1895. [5] Archiv. Internat. de Lar. et de Rhin. vii., 4, July-Aug., 1894.

## Figures and Tables

**Fig. 1. f1:**
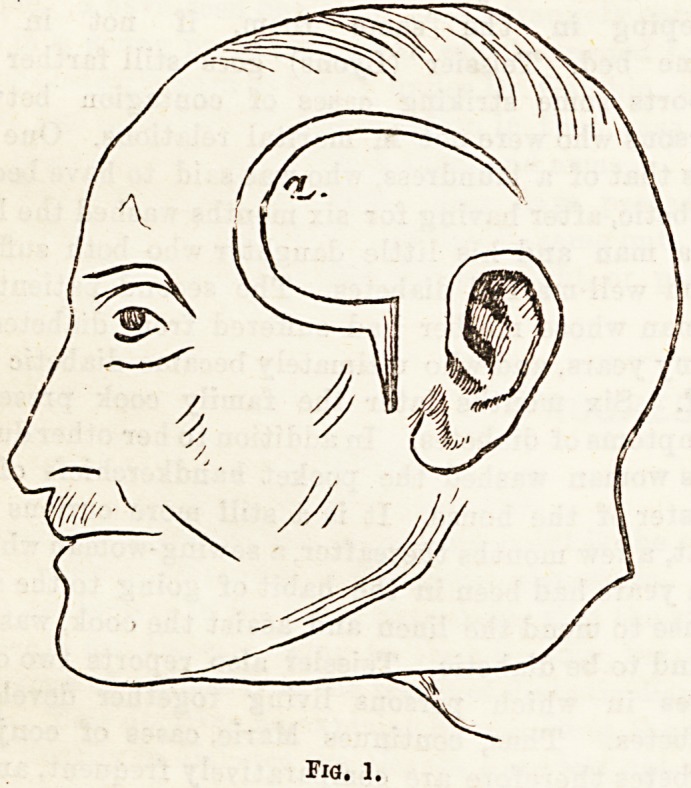


**Fig. 2. f2:**